# PRC2 Diversity in Neuronal Differentiation and Developmental Disorders

**DOI:** 10.3390/genes16101191

**Published:** 2025-10-13

**Authors:** Jasmine Akoto, Thomas Roule, Naiara Akizu

**Affiliations:** 1 Perelman Center for Cellular and Molecular Therapeutics and Center for Brain Research in Development, Genetics and Engineering, Children’s Hospital of Philadelphia, Philadelphia, PA 19104, USA; jasmine.akoto@pennmedicine.upenn.edu (J.A.); roulet@chop.edu (T.R.); 2 Department of Pathology and Laboratory Medicine and Genetics and Epigenetics Graduate Group, Perelman School of Medicine, University of Pennsylvania, Philadelphia, PA 19104, USA

**Keywords:** Polycomb, PRC2, EZH1, EZH2, epigenetics, neurodevelopmental disorders, Autism, intellectual disability, overgrowth, chromatin, neural stem cells, neurogenesis

## Abstract

Advances in genetic studies have not only improved the diagnosis and treatment of neurodevelopmental disorders but also uncovered human-specific aspects of nervous system development. The generation of neuronal diversity in the human brain relies on tightly regulated epigenetic mechanisms, with Polycomb Repressive Complex 2 (PRC2) emerging as a key player. In this review, we first summarize foundational studies that established the role of PRC2 in the epigenetic maintenance of transcriptional silencing. We then highlight recent insights into the increasing evolutionary complexity of PRC2 subcomplexes, their roles in neurodevelopment, and their contribution to human developmental disorders.

## 1. Introduction

Neuronal differentiation, similar to other differentiation processes, entails the progressive commitment of neural precursor cells, followed by the generation of newborn neuronal subtypes and their functional maturation [1]. Two major events define this process: the restriction of differentiation potential and the acquisition of neuronal type identity and function. While changes in gene expression govern the establishment of neuronal identity and function, the commitment to specific neural lineages and the preservation of their identity requires the maintenance of established gene expression patterns for long periods [2,3]. A classic mechanism to preserve the repression of genes associated with alternate cell lineages involves the trimethylation of lysine 27 on histone H3 (H3K27me3) of chromatin.

Originally discovered in *Drosophila melanogaster* as gatekeepers of body segmentation, Polycomb Group (PcG) proteins are central to H3K27me3 deposition and the long-term maintenance of gene repression [4]. To execute their function, PcG proteins assemble into Polycomb Repressive Complex 1 and 2 (PRC1 and PRC2). While the core components and function are evolutionarily conserved, PcG subcomplexes have diversified extensively in their composition, chromatin-targeting mechanisms, and biological functions, particularly in more complex organisms. For example, while Drosophila has two PRC1 and one PRC2 complexes, mammals have at least six PRC1 and two PRC2 variants [5]. Emerging evidence suggests that this diversification enables context-specific genome regulation, essential for the expansion of cellular heterogeneity. Accordingly, distinct PRC1/2 subunits in mammals show cell type-specific expression [6,7,8] or division of catalytic activities [9]. However, despite the extensive literature defining the molecular function and expression pattern of each complex, the mechanisms leading to their functional specificity across cell types are not well understood.

In this review, we present a comprehensive discussion aimed at unraveling and interpreting our current understanding of the diversification of the Polycomb complexes, with a particular emphasis on PRC2 and neuronal cell type diversity. We first briefly review historic events that uncovered the basics of PcG- and H3K27me3-mediated epigenetic maintenance of gene repression. We then provide an update on the evolutionary diversification of PRC2 core and accessory subunits and their molecular roles. Finally, we discuss emerging evidence supporting context-specific functions of PRC2 subcomplexes from the perspective of neural development and developmental disorders.

## 2. The Discovery of Polycomb Group (PcG) Proteins as Epigenetic Guardians of Gene Repression

Nearly a century ago, Pamela Lewis coined the term “Polycomb” to describe the phenotype of mutant *Drosophila melanogaster* flies exhibiting extra sex combs [10]. In subsequent work, Ed Lewis noticed that *Polycomb* (*Pc*) mutant flies resembled flies ectopically expressing Hox genes, a group of homeotic genes that provide antero-posterior identity to body segments [11]. Like Hox mutants, *Pc* flies showed body parts misplaced along the antero-posterior axis. This groundbreaking observation suggested that the product of the *Pc* gene was necessary to repress Hox genes [11]. The discovery of additional genes whose mutations caused similar body transformations led to defining them as PcG genes [12]. Among these, *extra sex combs* (*esc*), a founding PcG member with *Pc*, became of particular importance given that fly embryos lacking both maternal and zygotic *esc* displayed ectopic Hox gene expression only after gastrulation. This phenomenon revealed that ESC was required for maintaining, but not for establishing, Hox gene repression [13]. Furthermore, the phenotypic comparisons with *Pc* mutants suggested a mechanism where ESC would be required prior to PC for maintaining the correct Hox gene expression and body segmentation [13,14]. These studies provided the first mechanistic evidence of PcG-mediated maintenance of Hox repression and positioned PcG proteins as key components of the epigenetic cellular memory system.

## 3. Diversification of PRC2 Composition

The end of the 20th century was marked by the emergence of epigenetics as a field focused on studying mechanisms that preserve gene expression programs over time and cell divisions [15,16,17]. Chromatin, the multimeric complex formed by repeating units of DNA wrapped around an octamer of histone proteins, was increasingly recognized as a central platform for the inheritance of gene activity information not encoded in the genome [18]. As PC and other PcG proteins were being found to be associated with chromatin through then-pioneering chromatin immunoprecipitation assays [19,20] and in vitro histone binding experiments [21], it became clear that chromatin played a role in how PcG proteins maintain gene repression patterns established by transcription factors early in development. However, it was not until the discovery of three key pieces of evidence that the PcG-mediated repression mechanism emerged. First, purification of PcG proteins from fly embryos revealed two distinct complexes, one containing PC as a core member and designated PRC1 [22], and the other, PRC2, formed by the interaction of ESC and another PcG protein known as Enhancer of Zeste (E(Z)) [23,24]. Second, PRC1 binding to chromatin was found to depend on E(Z), despite the lack of physical interaction between them [25,26]. Finally, the identification of histone methyltransferase activity in E(Z) led to what is now recognized as the classical PcG-mediated epigenetic silencing mechanism [27,28,29]. Under this classical model, E(Z) and its mammalian homolog EZH2 catalyze H3K27me3. This histone covalent modification is recognized by the chromodomain of PC (or mammalian homologs, CBX) and recruits PRC1 to chromatin [27]. The sequential recruitment of PRC2 and PRC1 to chromatin triggers structural changes that contribute to maintaining the repression of targeted genes [30].

The classical model of PcG recruitment and repressive domain maintenance prevailed for over a decade, but recent discoveries have revealed a far more complex mechanism. This complexity was first highlighted by the finding that the core PRC1 subunits, RING1A/B, catalyze mono-ubiquitination of histone H2A at lysine 119 (H2AK119ub) [31,32], prompting a revision of the classical view. Just like PRC1 binding to PRC2-catalyzed H3K27me3, PRC2 can also bind to PRC1-catalyzed H2AK119ub [33,34]. These reciprocal interactions generate a positive feedback loop that propagates PRC1 and PRC2 recruitment and sustains repressive chromatin domains across space and time [35,36,37]. Emerging data further indicate that the interplay and activity of PRC1 and PRC2 vary depending on their subunit composition and spatiotemporal context [38,39,40,41,42]. Although, together, these findings illustrate that PcG-mediated repression is orchestrated by multiple, interconnected layers of regulation, the silencing mechanism remains incompletely understood. Recruitment can be influenced by chromatin modifications as well as interactions with RNA molecules, while mechanisms contributing to repression range from chromatin compaction to interference with transcription factors and the RNA polymerase. Since the complexity of these mechanisms, particularly those involving PRC1 variants and their diverse functions, have been extensively reviewed elsewhere [5,30,43,44], here we focus on PRC2 variants, emphasizing insights from developmental models and genetic neurodevelopmental disorders that reveal potential divergences.

**Figure 1:**
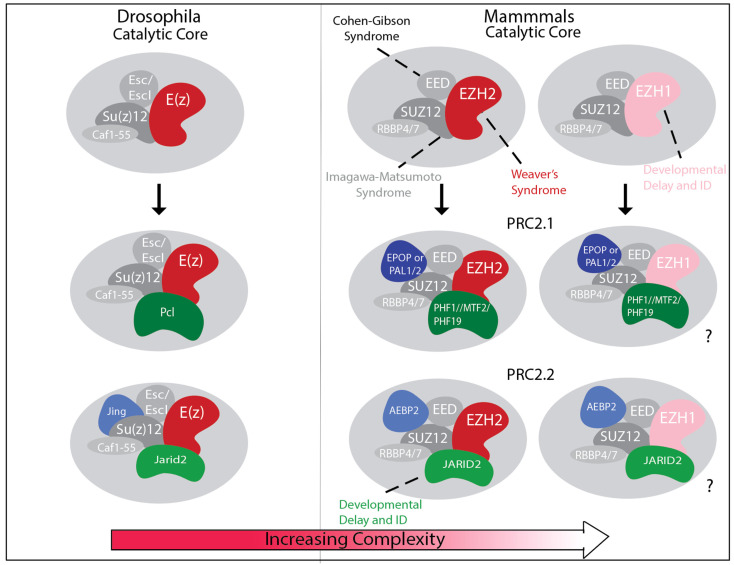
Graphical representation of PRC2 core and accessory subunits depicting increased number of PRC2 subcomplexes as cellular diversity increases from Drosophila to mammals. (**Left**) Drosophila PRC2 consists of the core subunits Enhancer of Zeste (E(Z)), Extra Sex Combs (ESC) or Extra Sex Combs like (ESCL), Suppressor of Zeste-12 (SU(Z)12), and CAF1-55, and either the accessory subunit Polycomb-like (PCL) or JING and JARID2. (**Right**) In mammals, the core PRC2 is formed by either EZH1 or EZH2 (E(Z) homologs), Embryonic Ectoderm Development (EED) (ESC homolog), Suppressor of Zeste 12 Protein Homolog (SUZ12) (SU(Z)12 homolog), and RBBP4 or 7 (CAF1-55 homologs). In addition, the interaction with either one of the three homologs of Drosophila PCL protein, PHF1, MTF2, or PHF19, and either PALI1, PALI2 (PRC2 Associated LCOR Isoform paralogs), or EPOP (Elongin BC complex and the PRC2-associated Protein) forms PRC2.1 subcomplexes, whereas the interaction with Adipocyte Enhancer-Binding Protein 2 (AEBP2) and Jumonji, AT-rich interaction domain 2 (JARID2) creates PRC2.2 subcomplexes. “?” indicates the uncertainty about PRC2.1 and PRC2.2 composed with EZH1, due to limited supporting literature. Mutations in nearly all core subunits (EZH1, EZH2, EED, and SUZ12) and an accessory subunit (JARID2), as indicated in the figure, are associated with human neurodevelopmental disorders.

The core PRC2 in mammals is composed of four proteins: EED, SUZ12, RBBP4 or RBBP7, and either EZH1 or EZH2. In addition, selective interactions with different accessory proteins define at least two major PRC2 subcomplexes with distinct activities: PRC2.1, which is formed by the interaction of the core with PHF1, MTF2, or PHF19, together with either PALI1, PALI2, or EPOP; and PRC2.2, which includes JARID2 and AEBP2 instead [40,41,42] (Figure 1). Evidence indicates that PRC2.1 facilitates the recruitment of the complex to unmethylated CpG reach islands (CGIs) devoid of activating transcription factor motifs for the establishment of H3K27me3 repressive domains in developmental gene promoters [45,46]. Accordingly, deletion of *Mtf2* in mouse embryonic stem cells significantly impairs de novo H3K27me3 deposition and spreading [45]. In contrast, the ability of JARID2 and AEBP2 to bind to H2AK119ub enables the allosteric activation of PRC2.2 to propagate repressive domains at loci previously targeted by PRC1 [35,38,39]. The latest recruitment sequence is particularly important for PRC2 targeting to inactive X chromosomes [47], but also for maintaining Polycomb repressive domains at developmental genes, as demonstrated by loss of PRC2.2 and H3K27me3, and reactivation of target genes, upon the depletion of PRC1 ubiquitin-transferase activity [36,37]. Despite their functional differences, however, the coordination of PRC2.1 and PRC2.2 with PRC1 is critical for the spatiotemporal regulation of developmental gene expression programs during cell fate specification and differentiation.

Homologs of most accessory proteins defining PRC2.1 and PRC2.2 in mammals have also been identified in Drosophila [48,49] (Figure 1), suggesting that ‘division of labor’ across PRC2 subcomplexes is an essential feature conserved throughout evolution. However, the presence of many more paralogs for most PRC2 subunits in mammals raises outstanding questions that remain unknown. For example, are different paralogs within PRC2.1 and PRC2.2 interchangeable or do they further expand the number of functionally distinct PRC2 subcomplexes? Is the evolutionary diversification of PRC2 subcomplexes associated with the expanded cellular diversity in more complex organisms? Current studies, largely focused on proliferating cancer cells or pluripotent stem cell models, fail to capture the full spectrum of cellular diversity and stochiometric variation in PRC2 subcomplexes. As a result, the functional contributions of PRC2 subcomplexes, especially those that are underrepresented in commonly used models, is largely unexplored. Emerging research in human developmental models and the growing number of neurodevelopmental disorders (NDDs) caused by pathogenic variants in genes encoding core and accessory PRC2 subunits offer powerful opportunities to shed light on how PRC2 subcomplexes contribute to cellular diversity.

## 4. Context-Specific Functions of PRC2: Lessons from Development and Neurodevelopmental Disorders

NDDs are a group of conditions characterized by a spectrum of motor, language, cognitive, and social behavioral defects, often associated with risk variants in genes expressed during early mid-gestation of human brain development [50,51]. This developmental window coincides with a shift from proliferative to neurogenic divisions of cortical neural stem cells (e.g., radial glia (RG)), generating intermediate neural progenitors with progressively restricted lineage potential that give rise to different types of excitatory and inhibitory neurons [1]. A rapidly growing number of studies, enabled by human pluripotent stem cell (hPSC)-derived brain organoid technologies, suggest that disrupted or uncoordinated neuronal subtype generation is common to NDD pathogenesis [52,53]. Chromatin plays a critical role coordinating neuronal subtype generation by determining how accessible developmental genes are to external cues. Indeed, a recent study demonstrated that transient inhibition of specific chromatin-modifying programs in neural progenitor cultures can override the intrinsic barriers that establish the long maturation pace of human neurons [54]. In line with these studies, variants in genes encoding chromatin modifiers are among the most common genetic causes of NDDs [51,55,56,57].

Mutations in core and accessory PRC2 subunits are linked to diverse NDDs [6,58,59] (Figure 1 and Table 1). However, clinical features vary depending on the mutated subunit, suggesting context-specific functions rather than being interchangeable. The co-evolution of expanded PRC2 subcomplexes alongside increasing cellular diversity also supports this idea. For example, only focusing on the central nervous system, the adult Drosophila brain contains ~140,000 neurons of 10,000 types [60], whereas an adult human brain has ~100 billion neurons and likely millions of types [54,61]. In parallel, PRC2 subcomplexes have increased from 2 in Drosophila to potentially 12 in humans.

**Figure 2:**
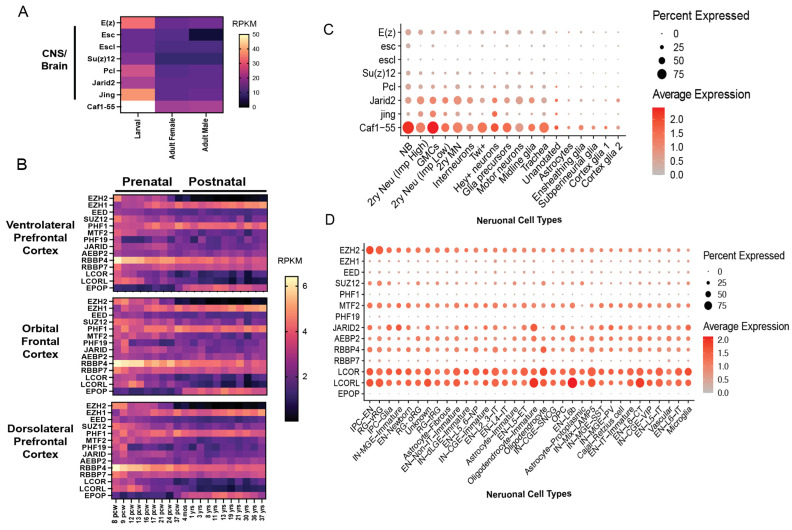
Expression of PRC2 subunits in Drosophila and Human developing and adult nervous system. (**A**) Heatmap displaying the expression levels (RPKM) of Polycomb Group (PcG) genes in the central nervous system of third-instar (L3) larvae and the brain of adult males and females of Drosophila. Data was obtained from FlyAtlas2 Anatomical Expression Data. (**B**) Heatmaps displaying the expression levels (RPKM) of mammalian PRC2 core and accessory subunits during prenatal and postnatal brain development across different regions. Data were obtained from mRNA-sequencing datasets in BrainSpan. The average expression value was calculated for each time point in each brain region. (**C**) Dot plot showing scRNAseq expression of PcG genes across neuronal cell types in the Drosophila third-instar larval ventral nerve cord (VNC). (**D**) Dot plot showing scRNAseq expression of PRC2 genes across neuronal cell types in second-trimester developing human neocortex. Dot size indicates the percentage of cells expressing each gene, and color the average expression level. Dot plots were generated using the Seurat R package (v4.3.0) with the DotPlot function, based on author-defined cell type annotations and normalized RNA data from the original study. Note that *LCOR* and *LCORL* are the genes encoding PALI1 and PALI2. Abbreviations: NB, neuroblast; GMC, ganglion mother cell; Imp, insulin-like growth factor 2 mRNA-binding protein; RG, radial glia (vRG, ventricular RG; tRG, truncated RG; oRG, outer RG); OPC, oligodendrocyte precursor cell; IPC, intermediate progenitor cell (IPC–EN, excitatory neuron progenitor; IPC–Glia, glial progenitor); IN, interneuron (dLGE, dorsal lateral ganglionic eminence; MGE, medial ganglionic eminence; CGE, caudal ganglionic eminence; SST, somatostatin; PV, parvalbumin; VIP, vasoactive intestinal peptide; SNCG, synuclein gamma; EN, excitatory neuron (IT, intratelencephalic; ET, extratelencephalic; CT, corticothalamic); NP, near-progenitor.

Consistent with a role for PRC2 diversification in supporting cellular complexity during evolution, PRC2 subunit expression differs across neural types and developmental stages, particularly in humans, compared with flies. As illustrated by the analysis of publicly available expression datasets (Figure 2A–C), in Drosophila, the expression of most PRC2 subunits peaks in the larval central nervous system (CNS) (Figure 2A). Similarly, in humans, the expression of *EZH2* and most core and accessory subunits are high prenatally and decline after birth in the cerebral cortex. In contrast, *EPOP* is enriched postnatally, while *EZH1* and *PHF1* increase at 16–20 postconception weeks, coinciding with the onset of cortical neurogenesis, when neural stem cells transition from proliferative to neurogenic divisions (Figure 2B), and remain constant afterward.

Although bulk PRC2 subunit expression patterns are similar across different regions of the human cerebral cortex (Figure 2B), single-cell RNA sequencing data reveals neural type-specific differences, which are more pronounced in the human second trimester cortex [62] than in Drosophila third-instar larval ventral nerve cord (VNC) [63] (Figure 2C,D). In Drosophila, all PRC2 subunit expression is highest in proliferating neuroblasts (NBs) (Figure 2C). By contrast, in the developing human brain, expression patterns are more variable (Figure 2D). *EZH2* and *SUZ12* are enriched in neural precursor cells, including radial glia (RG) and intermediate neural progenitor cells (IPCs), compared with postmitotic neurons. *EZH1* and *EED* show relatively uniform expression across proliferating and postmitotic neural populations, with slightly higher levels in excitatory neurons of layer 2 and 3 and select interneurons. At this developmental stage, PRC2.1 subunits *MTF2* and *LCOR/LCORL* (which encode PAL1/2) show a variable expression across different neural types but predominate over *EPOP* and *PHF1/19*, which may be more prominent in postnatal populations, as suggested by the bulk RNA seq data in Figure 2B. PRC2.2 subunits *AEBP2* and *JARID2* also display variable cell type expression, though not always proportional to each other, implying the existence of possible PRC2 alternative subcomplexes or independent roles of these subunits. While the stoichiometry of PRC2 subcomplexes across developmental stages and neural cell types remains to be defined at protein level, these findings highlight unique aspects of PRC2 function in humans. Such differences align with the distinct clinical phenotypes arising from mutations in individual PRC2 subunits discussed below.

### 4.1. EZH2, EED and SUZ12 Associated Overgrowth with Intellectual Disability (OGID) Syndromes

While the role of somatic PRC2 dysfunction in cancer is well established, the connection between germline mutations in PRC2 subunits and NDDs only began to emerge in 2012, with the discovery of de novo *EZH2* variants as one of the first causes of an OGID syndrome [64,65]. The syndrome had long been clinically recognized as Weaver syndrome (WVS, OMIM #277590) by its characteristic prenatal overgrowth persisting postnatally as tall stature and advanced bone age, varying degrees of intellectual disability, psychomotor delay, and a distinctive facial appearance including macrocephaly, eyelids that slant down, and large or low-set ears [66]. Most individuals with WVS carry heterozygous missense mutations that show reduced histone methyltransferase activity and H3K27me3 levels when tested in vitro or in cells [67,68,69]. Notably, few of these mutations have also been detected as somatically acquired mutations in cancers (e.g., EZH2 NM_004456: p.D664V, p.R684C and p.Y733*), and consistently, tumors, such as neuroblastoma, have been detected in some WVS patients [65,69].

A smaller subset of WVS patients harbor heterozygous truncating mutations located in the last exon, or partial or full gene deletions consistent with haploinsufficiency as the underlaying genetic mechanism [58]. However, recent work shows that certain WVS-associated *EZH2* variants abolish nearly all H3K27me3 when expressed in *Ezh2* heterozygous-null mouse ESC, indicating a dominant negative effect. Notably, some of these dominant variants (e.g., p.E745K, p.R684C and p.Y733*) have been identified in a subset of WVS showing polymicrogyria (i.e., excessive cortical folding) on brain imaging, suggesting they may alter specific EZH2 functions impacting cortical neuron migration [69,70,71,72]. Furthermore, a gain-of-function variant (p.A738T) that increases catalytic activity has also been identified in a patient with WVS facial features but growth restriction instead of overgrowth [73]. These findings underscore the importance of functionally assessing genetic variants, not only to elucidate mechanisms of inter-individual differences in clinical manifestations and severity, but also to inform therapeutic strategies.

Although no effective treatment currently exists for WVS, the large repertoire of pharmacological compounds developed to target chromatin regulators involved in cancer provides potential therapeutic avenues for managing progressive symptoms in WVS, including postnatal overgrowth. However, while EZH2 inhibitors may benefit patients with gain-of-function mutations, individuals with typical WVS caused by loss-of-function or dominant-negative *EZH2* variants may require alternative approaches, such as inhibitors of H3K27me3 demethylases KDM6A and KDM6B. Indeed, treating a mouse model of WVS expressing the EZH2 p.R684C allele with GSK-J4, a dual KDM6A/B inhibitor, reverses osteogenic defects that lead to overgrowth [74]. Beyond pharmacological strategies, a recent proof-of-concept study has also explored CRISPR-based correction of *EZH2* mutations in humanized *Ezh2* mouse cells [75], highlighting the future potential of genetic medicine for WVS.

As EZH2 methyltransferase activity depends on the assembly into the PRC2 core complex with EED and SUZ12, it is not surprising to find children with OGID carrying mutations in *EED* and *SUZ12,* although these are rarer than WVS-associated *EZH2* mutations [76,77]. De novo mutations in *EED* cause Cohen–Gibson syndrome (COGIS, OMIM #617561), which tends to be more severe than WVS. Of the COGIS patients reported to date, 100% show overgrowth and intellectual disability, compared with 93% and 85%, respectively, in WVS [76]. The majority of COGIS patients carry *EED* missense mutations, the functional consequences of which are challenging to predict. However, emerging data indicates that these mutations reduce PRC2 activity [9,78]. Indeed, a recent study noticed that COGIS-associated EED (NM_003797) mutations p.R236T and p.R302S/G alter amino acids implicated in the interaction with the histidine at position 129 of EZH2, and demonstrated that disrupting this interaction with pharmacologic compounds or even with the EZH2 p.H129C variant inhibits PRC2 activity [9].

Germline heterozygous *SUZ12* variants cause Imagawa–Matsumoto syndrome (IMMAS, OMIM#618786), which presents with less severe overgrowth and intellectual disability than WVS and COGIS [76]. The predominance of truncating mutations that generate premature stop codons suggests that pathogenic variants cause partial or full *SUZ12* loss-of-function. Nevertheless, several missense mutations have also been identified, most clustering in the VEFS-box region of SUZ12 [76]. This region is critical for PRC2 assembly and the stabilization of catalytically active EZH2 conformation [79]. The inability of the SUZ12 (NM_015355) p.E610V missense variant to restore H3K27me3 levels in SUZ12 knockdown cells [78] further supports haploinsufficiency or dominant-negative effect as the genetic mechanisms of IMMAS and, more broadly, PRC2-associated OGID syndromes.

Numerous studies in the past decades have focused on elucidating the role of *EZH2*, *EED*, and *SUZ12* in development, even before their link with OGIDs. In most species, complete deletion of core PRC2 subunits and loss of H3K27me3 results in gastrulation defects that terminate embryonic development [80,81]. Therefore, animal models have been limited to tissue- or cell type-specific depletions, heterozygous-null alleles, or pathogenic variant expression. These strategies revealed, early on, that deletion of *Ezh2* from forebrain cortical neural stem cells, generated by crossing mice carrying floxed *Ezh2* allele with *Emx1-Cre*, advances the transition to basal neural progenitors and the timing of differentiation to upper layer neurons [82]. Unlike WVS patients, however, these mice exhibit thinner cerebral cortices and do not show cognitive behavioral defects or macrocephaly. Likewise, according to the Mouse Phenotyping Consortium, heterozygous deletion of *Ezh2* does not cause overgrowth or behavioral defects in mice (https://www.mousephenotype.org/data/genes/MGI:107940 (accessed on 10 September 2025), while *Suz12* haploinsufficiency causes brain malformations that are more severe than those observed in IMMAS [83]. In contrast, WVS mouse models that have been generated using CRISPR-mediated genome editing (EZH2 p.V626M and p.R684C) show overgrowth, but neurobehavioral defects and structural brain abnormalities have not been reported [68,74], similar to *Eed* heterozygous-null mice [84]. These data are consistent with genetic mechanisms that differ from haploinsufficiency as the cause of PRC2-associated overgrowth or a specie-specific role of PRC2 in development, particularly of the nervous system.

In mouse and human embryonic stem cells (ESCs), PRC2 targets many development regulator genes, including *NEUROG1/2*, *GATA4*, *OLIG1* transcription factors or members of the *WNT* and *BMP* signaling molecules [85,86,87,88]. These genes are lowly expressed or repressed in ESCs but poised for activation in a cell type-specific manner upon differentiation [85]. Comparison of mouse and human ESCs studies, however, also supports that developmental PRC2 function is evolutionarily distinct and context dependent.

In mouse ESCs, loss of PRC2 through *Eed* deletion derepresses developmental genes marked by H3K27me3 at their promoters, though pluripotency and self-renewal remain intact [85,89]. Likewise, upon neural induction in culture, PRC2-deficient ESCs fail to repress genes that specify non-neural lineages [85,90]. A different scenario applies to anterior neural development genes marked by PRC2 at poised enhancers instead of at promoters. While these genes remain inactive in *Eed*-null mouse ESCs, they also fail to activate upon neural induction due to a role of PRC2 in facilitating enhancer and promoter interaction necessary for RNA Polymerase II recruitment [91]. These data indicate that depending on the genomic element, PRC2 function is different, though required for a coordinated transition of gene expression programs during development [91]. Accordingly, a recent work has shown that, although mouse ESCs can self-renew and differentiate into all three germ layers in vitro, upon depletion of H3K27me3 using EZH2 inhibitors and dominant negative EZH2 variants, they fail to maintain lineage-specific expression programs, eventually leading to unstable differentiation states and cell death [92].

In contrast to mouse ESCs, human ESCs rely more heavily on PRC2. Loss of PRC2 not only derepresses developmental target genes but also compromises ESC self-renewal, driving spontaneous differentiation toward meso-endoderm fates [86,93,94]. These species-specific differences may reflect the distinct developmental states of cultured ESCs. While mouse ESCs are maintained in naïve state, human ESCs are generally primed. Naïve ESCs have broader differentiation potential than primed ESCs, and H3K27me3 levels are low and rarely enriched at developmental gene promoters [95,96]. Consequently, recent studies show that naïve human ESCs, similar to mouse ESCs, tolerate PRC2 loss better, although defects emerge in trophoblast genes and differentiation [86,93,94,96].

Collectively, these findings reveal fundamental differences in how PRC2 maintains transcriptional repression programs across species and developmental stages and underscore the importance of human-specific models to study the mechanisms of OGID syndromes.

### 4.2. EZH1 Gain- and Loss-of-Function Variants in Overlapping Neurodevelopmental Syndromes

The main difference between Drosophila and mammalian PRC2 lies in the evolutionary duplication of the catalytic subunit, *E(z)*: in mammals, PRC2 can be formed by either EZH2 or its paralog, EZH1 (Figure 1). Despite being the first *E(z)* homolog to be cloned, EZH1 has traditionally been considered the minor catalytic subunit, mainly due to its weaker histone lysine methyltransferase (HMT) activity. Indeed, its catalytic activity was only recognized six years after that of EZH2, following the development of more sensitive HMT assays [97,98,99]. Compared to EZH2, EZH1 is about ten times less catalytically active and primarily mono-methylates H3K27 rather than tri-methylating it [99]. Accordingly, in proliferating cells, EZH1 can sustain H3K27me1 after EZH2 depletion, but only partially maintains H3K27me2 and H3K27me3, whereas EZH2 alone can preserve all three H3K27 methylation states even without EZH1 [9,98,100]. However, although EZH2 has a dominant role in H3K27 methylation, EZH1 binds nucleosomes and compacts chromatin more strongly, owing to a unique patch of basic amino acids in its central disordered loop [101]. How this EZH1-mediated chromatin compaction contributes to developmental PRC2 function remains unclear, but one possibility is that it helps preserve repressed Polycomb domains in postmitotic cells, where EZH2 expression is minimal. Alternatively, EZH1 may facilitate the recently reported interactions between poised enhancers and cognate promoters for RNA polymerase recruitment and activation upon differentiation [91]. Indeed, evidence indicates that EZH1 is required for the transcriptional activation of muscle and blood differentiation genes [7,8].

Although EZH1 has historically remained in a secondary plane due to its apparent low molecular and disease relevance, emerging evidence challenges this view. In 2019, a genetic analysis of recessive intellectual disabilities (IDs) uncovered a homozygous truncating *EZH1* variant (NM_001991: p.Leu80Serfs*6) as the candidate cause of ID in two siblings born from healthy consanguineous parents [102]. Subsequent identification of ten additional patients from five independent families, all harboring biallelic truncating variants, confirmed EZH1 deficiency as the cause of a novel NDD [6]. In the same study, we also reported heterozygous missense *EZH1* variants (at least some arising de novo) in nine patients presenting a similar NDD characterized by mild to severe language, motor, and cognitive developmental delays, intellectual disability, and atypical facial features, including protruding forehead, deep-set eyes, and flattened mid-face [6]. Surprisingly, in vitro histone methyltransferase assays and H3K27me3 analyses in cells revealed that at least two missense variants (p.A678G and p.Q731E) increase catalytic activity of EZH1, indicating that both EZH1 gain- and loss-of-function can lead to comparable NDD phenotypes. Notably, the lack of overgrowth as a defining characteristic of these patients, and recently reported sisters with recessive EZH1 variants and NDD [103], distinguish EZH1-associated NDDs from other PRC2-related OGID syndromes, suggesting distinct biological roles for EZH1 and EZH2.

In line with a distinct function, *Ezh1* deletion in mice and zebrafishes has a minimal impact on embryonic development, in contrast to *Ezh2* deletion [104,105]. Furthermore, *Ezh1*-deficient mice show only modest transcriptional changes in adult medium spiny neurons [104], though they exhibit decreased prepulse inhibition (https://www.mousephenotype.org/data/genes/MGI:1097695 (accessed on 10 September 2025)), a behavioral phenotype commonly linked to neuropsychiatric conditions. In human ESC-derived brain organoids, EZH1 loss delays neurogenesis, and EZH1 gain-of-function leads to premature generation of SATB2+ cortical neurons, suggesting asynchronous development as a common neuropathological mechanism.

Some of the differences between EZH2 and EZH1 may stem from their distinct spatiotemporal expression patterns. For instance, *EZH1* is ubiquitously expressed and becomes the predominant paralog as *EZH2*, and to some extent *SUZ12* and *EED*, decline during development, when progenitors differentiate, including into neurons (Figure 2B,D). This developmental switch suggests that EZH1 may assume specialized roles once cells exit the cell cycle. One possibility is that EZH1 assembles into PRC2 complexes with a composition or activity distinct from EZH2. Adding to this complexity, PRC2 has the ability to dimerize [101,106], potentially expanding the possible functional PRC2 configurations depending on the relative abundance of PRC2–EZH1 and PRC2–EZH2 across cell types. Such variability may allow PRC2 to fine-tune chromatin regulation in a context-dependent manner, particularly in tissues with high cellular heterogeneity such as the brain. Therefore, defining how EZH1 contributes to this diversity and what roles it plays in postmitotic cells, such as neurons, represents an important area for future investigation.

### 4.3. JARID2-Associated Neurodevelopmental Syndrome

Among PRC2 accessory subunits, JARID2 is the only one associated with an NDD. Heterozygous copy number variants (CNVs) and point mutations in *JARID2* cause a clinically distinct syndrome characterized by variable developmental and intellectual disability with mild dysmorphic facies (DIDDF, OMIM #620098), such as high anterior hairline, broad forehead, deep set eyes, pressed nasal bridge, and full lips [59,107,108,109]. Other commonly reported clinical manifestations are hypotonia, autistic features, and aggressive behavior. Out of the 26 affected individuals reported to date, 10 have truncating variants, 11 carry large deletions involving at least one exon, 1 patient has a tandem duplication covering exons 1–7, and 4 have missense variants. While most of these mutations arose de novo, at least one case of inheritance from a mildly affected father to his son has been reported [59]. Given the prevalence of deletions and truncating *JARID2* variants, haploinsufficiency is the most likely genetic mechanisms of the disease. Consistently, loss-of-function variants are underrepresented in the ~800,000 genomes reported in gnomAD v4.1.0, leading to a pLI = 1 and pLoF o/e = 0.13 that support *JARID2* haploinsufficiency as pathogenic. Nonetheless, functional studies, particularly of missense variants carried in patients with apparently milder phenotypes, may reveal alternative genetic mechanisms in the future.

Like PRC2-EZH2 core subunits, *JARID2* is highly expressed during early development and declines as cells differentiate [110,111]. Consistently, JARID2 is essential for embryogenesis and tissue homeostasis [112]. Deletion of *jarid2* in Xenopus leads to gastrulation arrest, and knockout mouse embryos die at embryonic day 10.5–15.5 due to neural tube, heart, and hematopoietic defects that vary in severity depending on the genetic background of the mouse strain [113,114,115]. Data from mouse ESCs also support that in vitro differentiation is *Jarid2*-dosage sensitive given that homozygous and heterozygous *Jarid2* knockout mouse ESCs show delayed differentiation to the three germ layer derivatives, including neural lineages [116,117,118,119].

In contrast, little is known about the function of JARID2 in human neural development. A recent genomic characterization revealed that the widely used KOLF2.1J human induced pluripotent stem cell (iPSC) line harbors CNVs, including one leading to *JARID2* haploinsufficiency [120]. However, global pluripotency and neuronal differentiation appear largely preserved, although derived neurons show reduced excitatory postsynaptic activity [121]. Of note, the lack of overgrowth and macrocephaly in DIDDF patients suggest that JARID2 and/or JARID2-containing PRC2 subcomplexes have different molecular or cell type-specific functions compared to canonical PRC2-EZH2 complexes. Therefore, generating human models carrying patient mutations and proper controls will be critical to elucidating how JARID2 diversifies PRC2 functions and shapes human development.

### 4.4. PRC2 Subunits Not Yet Implicated in Human Neurodevelopmental Disease

Other accessory subunits, including the PCL homologues PHF1, MTF2, and PHF19, as well as PALI1/2 and EPOP, which define PRC2.1, and the PRC2.2 subunit AEBP2, have not yet been linked to neurodevelopmental disorders. Nevertheless, studies with developmental models suggest that each of these factors may play distinct roles in development. For example, *Phf1*- and *Phf19*-deficient mice are viable and lack overt neurobehavioral abnormalities (https://www.mousephenotype.org/data/genes/MGI:98647 (accessed on 15 September 2025) and https://www.mousephenotype.org/data/genes/MGI:1921266 (accessed on 10 September 2025)), whereas *Mtf2* deficient mice, which die before weaning age, show posterior skeletal defects consistent with anteroposterior body pattern transformations due to Hox gene dysregulation typical of PcG proteins [122]. In addition, *Lcorl* deletion in mice causes increased aggression, hyperactivity, and pre-weaning lethality with incomplete penetrance (https://www.mousephenotype.org/data/genes/MGI:2651932 (accessed on 10 September 2025)).

Similarly to *Jarid2*, *Aebp2* deficiency leads to embryonic lethality and skeletal abnormalities in mice [123,124]. However, heterozygous *Aebp2*-null mice often exhibit features of abnormal neural crest development, including enlarged colons and hypopigmentation reminiscent of Hirschsprung disease and Waardenburg syndrome [124]. The lack of overgrowth in these mice is reminiscent of individuals with JARID2-associated DIDDF, further suggesting that the PRC2.2 subcomplex provides specialized functions to PRC2. In agreement with this, a recent study showed that genetically dissociating PRC2.2 in human PSCs increases genomic PRC2.1 occupancy [38]. Additionally, because PRC2.1 has higher affinity for chromatin binding, H3K27me3 levels at target genes were also increased, leading to functional consequences that are in opposition to PcG protein deletion [38].

Taken together, these findings suggest that individual PRC2 subcomplexes contribute unique regulatory functions, fine-tuning PRC2 activity in both temporal and cell type-specific manners. It remains to be determined whether these regulatory functions are intrinsic to each subcomplex or subunit or instead emerge from the relative abundance of different subcomplexes across cellular and genomic contexts. Addressing these questions will be key to understanding how PRC2 achieves functional diversity as cellular complexity increases.

## 5. Concluding Remarks

Advances in clinical genetics have not only improved the diagnosis of PRC2-associated NDDs but also uncovered human-specific aspects of PRC2 function. Distinct clinical features across PRC2-related syndromes and developmental models, together with spatiotemporal differences in subunit expression, point to cell type-specific roles likely shaped by variation in complex composition, an area that remains largely unexplored. In particular, the structure and function of PRC2 complexes in postmitotic cells and differentiating tissues, such as neurons in the human brain, are poorly understood. Integrating clinical genetic data with emerging human developmental models and increasingly sensitive proteomic approaches offers a powerful opportunity to elucidate how PRC2 diversity contributes to cellular specialization and functional complexity in humans.

## Figures and Tables

**Table 1:** Summary of PRC2 component function and mutant phenotype in Drosophila and mammals. N/A: no association.

**Drosophila**			
**Complex**	**Subunit**	**Molecular Function**	**Mutant Phenotype**
PRC2	E(z)	Catalyzes H3K27 methylation	Improper body segmentation; additional sex combs; lethality
PRC2	Esc/Escl	Facilitates histone methyltransferase activity	Improper body segmentation; additional sex combs; lethality
PRC2	Su(z)12	Facilitates histone methyltransferase activity	Improper body segmentation; additional sex combs; lethality
PRC2	Caf1-55	Enhances PRC2 activity	Lethality
PRC2.1	Pcl	Enhances the generation of H3K27me3 at target loci	Improper body segmentation; additional sex combs; lethality
PRC2.2	Jing	Modulates PRC2 methyltransferase activity	Homeotic transformations; Tracheal and nervous system defects; lethality
PRC2.2	Jarid2	Modulates PRC2 methyltransferase activity	Improper body segmentation; lethality
**Humans**			
**Complex**	**Subunit**	**Molecular Function**	**Neurodevelopmental Disease**
PRC2.1/PRC2.2	EZH2	Catalyzes H3K27 methylation	Weaver syndrome: overgrowth, moderate intellectual disability (in about 80% of patients), distinctive facial appearance with macrocephaly and occasionally polymicrogyria
PRC2.1/PRC2.2	EZH1	Catalyzes H3K27 methylation	NDD with developmental delay, mild-to severe intellectual disability and atypical facial features
PRC2.1/PRC2.2	EED	Facilitates and enhances H3K27 methylation and propagation	Cohen–Gibson Syndrome: Overgrowth, mild-to-severe intellectual disability (in 100% of patients), distinctive facial appearance with macrocephaly
PRC2.1/PRC2.2	SUZ12	Facilitates H3K27 methylation and stabilizes PRC2 complexes and interactions	Imagawa–Matsumoto syndrome: overgrowth, mild intellectual disability (in ~50% of patients).
PRC2.1/PRC2.2	RBBP4/7	Facilitates PRC2 recruitment to target loci	N/A
PRC2.1	PCL1/PHF1	Enhances H3K27 methylation and promotes recruitment to target loci	N/A
PRC2.1	PCL2/MTF2	Promotes recruitment to target loci and maintains H3K27 methylation	N/A
PRC2.1	PCL2/PHF19	Enhances H3K27 methylation and promotes recruitment to target loci	N/A
PRC2.1	EPOP	Modulates and promotes PRC2 methyltransferase activity	N/A
PRC2.1	PAL1/2	Modulates and promotes PRC2 methyltransferase activity	N/A
PRC2.2	JARID2	Facilitates recruitment of PRC2 to target loci and enhances deposition of H3K27 methylation	Developmental delay and intellectual disability with dysmorphic facies.
PRC2.2	AEBP2	Stabilizes PRC2 and helps stimulate H3K27 methylation activity	N/A

## Data Availability

The human brain gene expression data used in this study were obtained from the BrainSpan Atlas of the Developing Human Brain (https://www.brainspan.org/rnaseq/search/index.html (accessed on February 2025)). The Drosophila expression data were obtained from FlyBase (https://flybase.org/) under the FlyAtlas2 Anatomy RNA-seq dataset (accessed on August 2025). Dot plots showing scRNAseq expression of PcG genes across neuronal cell types in the Drosophila third-instar larval ventral nerve cord (VNC) were generated from the analysis of GEO: GSE235231 [63] and in human second trimester cortex from the analysis of the sn-multiomic dataset, available in the CELLxGENE archive (https://cellxgene.cziscience.com/collections/ad2149fc-19c5-41de-8cfe-44710fbada73 (accessed on August 2025)) [62].
